# Twenty years of activity at the European Institute of Oncology, Milan, Italy

**DOI:** 10.3332/ecancer.2014.ed37

**Published:** 2014-05-29

**Authors:** Linda Cairns

**Affiliations:** Science Editor, *e*cancermedicalscience, European Institute of Oncology, Milan, Italy

This year the European Institute of Oncology (IEO) celebrates its 20^th^ birthday. The institute, which was the brainchild of Professor Umberto Veronesi, is now one of the most important international multidisciplinary cancer centres. The hospital offers both public and private services and the research laboratories host international scientists who aim to identify mechanisms driving cells to become neoplastic and to develop therapies to counteract them.

The Scientific Committee boasts some of the most prestigious names in science and medicine including two Nobel Prize winners: Renato Dulbecco (Nobel Prize for Medicine 1975) and J Michael Bishop (Nobel Prize for Medicine 1989). Over the past 20 years many seminal papers from clinicians and research scientists at the IEO have paved the way for changes in clinical practice or have identified novel therapeutic targets or prognostic biomarkers.

IEO’s Palliative Care Service is committed to relieving suffering and improving quality of life for patients throughout the course of their cancer treatment. In 1999 the project “Ospedale senza Dolore” or “Hospital without Pain” was initiated.

IEO started the first clinical trial in Italy for the early diagnosis of pulmonary tumours ‘COSMOS’ (Continuous Observation of Smoking Subjects), involving over twenty-one thousand smokers who, once a year for five years, undergo a spiral CT scan. This test is able to reduce the mortality associated with this type of cancer by identifying tumours at an initial stage, when the possibility for cure is high.

In 2001 the IEO opened the first centre for integrated cancer diagnosis for women. This centre, located in the centre of Milan, offers the most advanced technological tests for the prevention and early detection of female cancers. In 2002 the IEO was accredited by the American Joint Commission, the agency that accredits hospitals in North America according to the highest standards of quality in existing health. IEO was the first non-US cancer centre to obtain the prestigious accreditation.

Following publications by Veronesi in the Lancet [1] and NEJM [2], the sentinel node technique is internationally recognized as a standard practice to detect the spread of breast cancer in the presence of small nodules. This innovative technique has proven to be of great value for patients with breast cancer and has spared thousands of women unnecessary axillary lymph node dissection.

Radiotherapy has always been a strong point of the institute and techniques such as 3D conformal beam radiation therapy, peptide receptor radiotherapy and Intraoperative Radiation Therapy (IORT). In 2012 the Advanced Radiotherapy Centre was inaugurated and this now ranks among the top 5 in the world. The centre has all the latest technology and can treat up to 4,500 patients a year.

In 2007, IEO introduced the da Vinci surgical system – a robot that has revolutionised patient treatment by making it possible for surgeons to make microscopic incisions with greater accuracy and control than ever before. The first application was for the prostate and the results were excellent with fewer side effects, less pain and quicker recovery. The use of robots was extended immediately to the surgical treatment of certain gynecological cancers, gastrointestinal and lung.

Patients at the IEO benefit from the experience and expertise of an internationally renowned team of pathologists, who see a greater percentage of unusual tumor specimens in a week than most pathologists see in a year. The Department of Pathology also acts as the reference laboratory for many international clinical trials for breast cancer patients, performing central re-assessment of the histological and biological characteristics of tumours. Modern platforms for automated immunohistochemistry, *in situ* hybridization and mutational analyses are all available.

In 2010 the IEO 2 Day Center opened, encompassing the modern idea of a hospital in which the diagnostic activity is separated from the therapeutic, both for greater efficiency and for quality of life of the patient.

The IEO today uses evidence-based medicine to treat patients and takes into account the effectiveness of the therapy while never forgetting the quality of life during and after treatments. Discoveries made over the last 20 years have improved and will continue to improve clinical practice and have opened up new frontiers in molecular research.
